# Two-Tier PSO Based Data Routing Employing Bayesian Compressive Sensing in Underwater Sensor Networks

**DOI:** 10.3390/s20205961

**Published:** 2020-10-21

**Authors:** Xuechen Chen, Wenjun Xiong, Sheng Chu

**Affiliations:** 1 School of Computer Science and Engineering, Central South University, Changsha 410083, China; 2 School of Electronics and Information Technology, Sun Yat-Sen University, Guangzhou 510275, China; xiongwj3@mail2.sysu.edu.cn (W.X.); chusheng@mail.sysu.edu.cn (S.C.)

**Keywords:** Bayesian compressive sensing, particle swarm optimization, three dimensional underwater wireless sensor network, Bayesian Crame´r-Rao Bound

## Abstract

Underwater acoustic sensor networks play an important role in assisting humans to explore information under the sea. In this work, we consider the combination of sensor selection and data routing in three dimensional underwater wireless sensor networks based on Bayesian compressive sensing and particle swarm optimization. The algorithm we proposed is a two-tier PSO approach. In the first tier, a PSO-based clustering protocol is proposed to synthetically consider the energy consumption and uniformity of cluster head distribution. Then in the second tier, a PSO-based routing protocol is proposed to implement inner-cluster one-hop routing and outer-cluster multi-hop routing. The nodes selected to constitute *i*-th effective routing path decide which positions in the *i*-th row of the measurement matrix are nonzero. As a result, in this tier the protocol comprehensively considers energy efficiency, network balance and data recovery quality. The Bayesian Cramér-Rao Bound (BCRB) in such a case is analyzed and added in the fitness function to monitor the mean square error of the reconstructed signal. The experimental results validate that our algorithm maintains a longer life time and postpones the appearance of the first dead node while keeps the reconstruction error lower compared with the cutting-edge algorithms which are also based on distributed multi-hop compressive sensing approaches.

## 1. Introduction

### 1.1. Motivation

In recent decades, the research on wireless sensor networks (WSNs) has been a hotspot for numerous applications, such as, environment monitoring [1], smart cities [2,3], military monitoring [4], maritime resource gathering [5] and health-care surveillance [6].

As maritime rights and interests are paid more and more attention, underwater wireless sensor networks (UWSNs), an important extension of WSNs into ocean, is of great value in various application fields, such as, oceanographic information collection, hydrological and environmental monitoring, resources exploration, disaster forecast, underwater navigation, military defense, and etc. [7]. For UWSNs, there exist two kinds of topological structures, two dimensional network and three dimensional network. In this work, we study the three dimensional UWSNs, with which all nodes are deployed at different depths in the ocean.

Generally, in WSNs, including UWSNs, the information from all sensor nodes, which are equipped with limited power battery, are gathered to the sink node. When the power of the node is used up, the node expires. While most nodes died, the sink node is no longer able to gain necessary information from common nodes. Considering the fact that the nodes are hard to recharge, energy consumption remains a key factor that affects the whole network lifetime. On the other hand, in underwater scenario, as the network usually occupies large area and the effective transmission semidiameter is limited, those nodes who might not transfer the signal to sink node in one hop will need relay nodes. As a result, to design an efficient data routing algorithm for UWSN, it is vital to reduce the processing complexity and traffic overhead and meanwhile ensure that the data are collected efficiently. Wang et al. [8] render a layering algorithm which divides nodes to different parts according to their depth and data are forwarding based on opportunistic directional forwarding strategy. Guan et al. [9] improved opportunistic routing by adding distance-vector. The algorithm uses a query mechanism to establish the distance vectors for sensor nodes, then opportunistic routing is developed to forward packet based on the distance vectors. Further studies tend to cluster all nodes in the network, and only transmit the merged message in the cluster head to the sink node. Common nodes just need to transmit their data to their cluster heads (CHs) in one hop. For instance, clustering is added as one stage in [10] to save energy better. This strategy cuts down the energy-consumption efficiently. In [11], clustering and CH transmission are introduced as well. After the formation of cluster and selection of CH, the courier nodes are deployed into the clusters to collect the aggregated data from the CHs, and the visiting tour is scheduled based on the Decisional Welzl’s algorithm.

However, the works above do not consider the facts that in UWSN, while the sensor nodes are deployed to measure environmental data, for example, maritime temperatures, the signals they collect usually possess spatial correlations that induce sparsity into practical application. Based on such a fact, we comprehensively consider the routing strategy and the data sparsity to save energy even further. In such contexts, compressive sensing (CS) is adopted to reduce communication cost while also preserve information precisely. In this paper, we propose a CS based schedule which simultaneously considers routing and nodes selection for overall data reconstructions at the sink node for 3-D UWSNs. We use Particle Swarm Optimization (PSO) to optimize both the clustering and routing protocols design while the factors consisting of fitness functions consist of signal recovery quality, energy consumption, network balance and etc.

### 1.2. Related Work

CS, which unifies sampling and compression, has been enthusiastically promoted and studied [12,13]. It states that when the original signal is sparse itself or sparse on some basis, we can reconstruct it from compressed measurements. Making use of its advantages on simple encoding and efficient compressing, many researchers apply CS in WSN to achieve advanced compression compared with conventional compression algorithms [14,15,16]. CS could also be utilized for sensor selection; that is, only part of nodes will be chosen to transmit the product of its data with a random coefficient to the sink [17,18,19]. In the context that all nodes transmitted their data directly to the sink node in one hop, Hwang et al. [20] propose both centralized and decentralized algorithms for sensor selection under multi-variated noise condition. In [21], a tree-based algorithm named CDG is designed to reduce the payload falling on nodes close to the BS. In [22,23], a WSN is partitioned into clusters. Sensor readings are sent to CHs and the CHs send the received data to the BS. However, the measurement matrices they adopt are all full Gaussian ones which consume more power than the sparse measurement matrix used in our work. In the other word, they do not combine the routing and nodes selection with the help of CS.

PSO is a computational method that can be used to improve the optimization of candidate solutions through iteration. The way to adopt PSO for data routing [24] has been studied in several literatures [25,26,27]. Ref. [25] proposes a novel bi-velocity discrete PSO approach which extends PSO from the continuous version to the binary or discrete domain to find routing paths for WSN. Tukisi et al. [26] presents a PSO based method to find routings for the network with energy harvesting. Targeting on the problem that some of the nodes would be left out during cluster formation, ref. [27] introduces to apply the concepts of PSO and gravitational search algorithm to prevent residual nodes. All of these studies consider the application of PSO either in the clustering or in the routing, i.e., only one tier. And they do not consider the application of CS.

To the best of our knowledge, there are few works besides the following literatures that have considered the application of CS into combination of data routing and nodes selection in WSNs, especially in UWSNs. In [28], the network is partitioned into clusters. Then each CH collects the sensor readings within its cluster and generates CS measurements to be forwarded directly to the base station. As a result, the overall CS measurement matrix at the base station is a block diagonal matrix. Later on, in [29], an improved method is introduced which allows the multi-hop routing of generated CS measurements through intermediate CHs. The authors generalize the two methods in [30]. Compared to our work, they obtain CS measurements in each CH and then forward to sink node while we postpone this operation to be done in the sink node to reduce the burden in CHs. On the other hand, while choosing multi-hop routing paths from the CH to the sink node, they use traditional tree-based algorithm while we design a PSO-optimization based scheme.Specifically targeting on three dimensional UWSNs as well, distributed multi-hop CS has been proposed by Gong et al. in [31]. This algorithm randomly chooses a subset with size *M* of *N* nodes in the first place, then each chosen node finds a tour to the sink node. During the traveling of the message through the tour, each node computes the product of its sensing data and a random weighted coefficient and adds the product to the intermediate result it received. Finally the sink node obtains *M* measurements and reconstructs the whole data.Compared to our work, they ignore the noise and select the nodes which participate in sensing by Bernoulli generator. To conclude, though these works use sparse measurement matrices similar to what we do, they only consider energy efficiency while choosing appropriate routs without considering the sensing quality. Furthermore, they do not consider the optimization of clustering.

In this work, we introduce a two-tier PSO protocol for both clustering and routing. Specifically, our contributions are listed as below.

(1) In the first tier, a PSO-based clustering protocol is proposed to find appropriate CHs with the comprehensive consideration of energy consumption efficiency and CH distribution uniformity.
(2) In the second tier, a PSO-based routing protocol is introduced to find appropriate routs. A routing path consists of inner-cluster one-hop routing and outer-cluster multi-hop routing and each path corresponds to one cluster. Hence the number of paths equals to the number of clusters. It is worth mention that the multi-hop routing in our scheme is not restricted to consisting of CHs only. Note that these paths correspond to the rows of the measurement matrix for CS. In this way, we implement the combination of routing and nodes selection based on CS. Hence, the fitness function of PSO-based routing protocol comprises of several factors, including the criterions for energy efficiency, network balance, and data recovery qualities. To measure network balance, we divide the nodes in the 3-D model into different layers in accordance with their horizontal distance to the sink node. As we adopt Bayesian CS (BCS), Bayesian Cramér-Rao Bound (BCRB) is added in the fitness function to reflect the recovery performance.
(3) With optimization in CH and routing chosen progress, the network could survive longer with relatively lower measurement error as demonstrated by the simulation results. What’s more, taking energy-balancing into consideration contributes to form a more balanced energy-consuming network every round cycle, and also prolongs the network lifetime and reduces the sensing error.

The remainder of this paper is organized as follows. In Section 2 we present preliminary knowledge. In Section 3, we introduce our system model for UWSN scenario. The detailed descriptions of the clustering and routing algorithms based on CS and PSO are given in Section 4. The simulation results are presented in Section 5. Finally, Section 6 concludes the paper, and introduces the future work.

The notation used in this paper is according to the convention. Symbols for matrices and vectors are in boldface.

## 2. Preliminary

### 2.1. Compressive Sensing

CS theory proves that if a signal z of dimension *N* is sufficiently sparse in a certain domain; that is z=Ψx and ${∥x∥}_{0}=K,K\ll N$, where $\Psi \in R^{N\times N}$ is an orthogonal basis and $x\in R^{N}$, it is efficient to recover z from a random measurement vector that is obtained by y=Φz. *M*, the length of y is much smaller than *N*. Φ is named as a measurement matrix or sensing matrix. In the sequel, the random non-zero coefficients in Φ obey Gaussian distributions.

In general, it is an ill-posed problem to recover signal z from y since y has much smaller dimensions than z. We have to minimize the solution’s $\ell_{0}$ norm to find the sparsest result. However, it is an NP-hard problem, which is not capable to be represented by mathematical formulas. Candés et al. [13] state that as long as a measurement matrix satisfies the restricted isometry property (RIP) condition, the $\ell_{0}$ norm can be replaced by $\ell_{1}$ norm. A measurement matrix satisfies RIP of order of *K* when

(1) $$(1-\epsilon ){∥x∥}_{2}\leq {∥\Phi \Psi x∥}_{2}\leq \left(1+\epsilon \right){∥x∥}_{2}$$

for all ${∥x∥}_{0}\leq 2K$ and some 0<ϵ<1. Afterwards, many methods for such a convex optimization problem were introduced to solve the $\ell_{1}$ norm optimization problem [32,33,34]. Later on, iterative-greedy-pursuit recovery algorithms [35,36,37] are proposed to reduce the time complexity.

### 2.2. Bayesian Estimation

In the presence of noise, the noisy measurement vector y is

(2) y=ΦΨx+n=Θx+n.

where $n\in R^{M}$ denotes the noise.

In the underwater acoustic channel, the overall noise at the receiver contains both the ambient noise and residual intersymbol interference (ISI) after equalization. Though the ambient noise is not AWGN in the underwater environment, the use of Gaussian approximation is often convenient and adequate [38]. In the presence of Gaussian ambient noise, according to [39] and [40], the overall noise can be treated as Gaussian, although it is not independent with regard to time step, since the signal after CS transform, i.e., transmitted signal is treated as Gaussian distributed. Hence, in the sequel, n is approximated as Gaussian distributed.

In [41], BCS is proposed to reconstruct a sparse signal from noisy measurements, if a statistical characterization of the signal is available. The computation time of BCS is comparable to or even faster than other classical greedy iteration algorithms. In this work, we adopt bayesian learning to recover the signal in the sink node. Therefore in this part, we make a brief review of BCS method.

$n_{i}$ indicates the *i*-th element of n in Equation (2), and is approximated as a zero mean Gaussian variable with variance $\sigma^{2}$. From the Bayesian point of view, to obtain the restoration of the original signal is to seek a full posterior probability function for **x**. Usually, a Laplacian distribution is a widely used prior for sparse signals. But considering it is not conjugate to Gaussian likelihood, in BCS, a hierarchical prior has been defined for **x**; that is, a zero-mean Gaussian prior is defined on each element of **x**

(3) $$p\left(x|\alpha \right)=\prod_{i=1}^{N}N(x_{i}|0,\alpha_{i}^{-1}),$$

where $\alpha_{i}$ is the inverse-variance of $x_{i}$ and α satisfies a Gamma prior with parameters *a* and *b* with $p\left(\alpha \right)=\prod_{i=1}^{N}\Gamma \left(\alpha_{i}|a,b\right)$. By marginalizing over the hyperparameter α, the overall prior is given by

(4) $$p\left(x|a,b\right)=\prod_{i=1}^{N}\int_{0}^{\infty }N\left(x_{i}|0,\alpha_{i}^{-1}\right)\Gamma \left(\alpha_{i}|a,b\right)d\alpha_{i}.$$

Likewise, the hyperparameter $\alpha_{0}=1/\sigma^{2}$ satisfies a Gamma prior $\Gamma (\alpha_{0}|c,d)$. Note that *a*, *b*, *c* and *d* are all set to zero to obtain a uniform hyperprior.

The goal of BCS is to estimate three parameters α, $\alpha_{0}$, and the most important one x. The posterior function of all unknowns is

(5) $$p\left(x,\alpha ,\alpha_{0}|y\right)=p\left(x|y,\alpha ,\alpha_{0}\right)p\left(\alpha ,\alpha_{0}|y\right).$$

The posterior of **x** is

(6) $$p\left(x|y,\alpha ,\alpha_{0}\right)={\left(2\pi \right)}^{-\frac{N}{2}}{\left|\Sigma \right|}^{-\frac{1}{2}}\exp\left(-\frac{1}{2}x-\mu^{T}\Sigma^{-1}x\mu \right),$$

with the mean matrix μ and the covariance matrix Σ,

(7) $$\begin{array}{cc} \mu & =\alpha_{0}\Sigma \Theta^{T}y\\ \Sigma & ={\left(\alpha_{0}\Theta^{T}\Theta +A^{-1}\right)}^{-1}. \end{array}$$

For the case of uniform hyperpriors,

(8) $$p\left(\alpha ,\alpha_{0}|y\right)\propto p\left(y|\alpha ,\alpha_{0}\right).$$

Finally, we can estimate all unknown parameters by maximizing the posterior in Equation (5). To find a approximate solution, we use EM algorithm to achieve the solution iteratively. In the algorithm, α and $\alpha_{0}$ are re-estimated by every iteration with the form

(9) $$\begin{array}{cc} \alpha_{i}^{new} & =\frac{\gamma_{i}}{\mu_{i}^{2}},\\ \frac{1}{\alpha_{0}^{new}} & =\frac{{∥y-\Phi \mu ∥}_{2}^{2}}{M-\underset{i}{\Sigma }\gamma_{i}}, \end{array}$$

where $\mu_{i}$ is the *i*th posterior mean weight from μ and $\gamma_{i}$ is defined as $\gamma_{i}≜1-\alpha_{i}\Sigma_{ii}$, with $\Sigma_{ii}$ the *i*-th diagonal element of the posterior covariance weight from Σ. Then new μ and Σ are obtained. The iterations terminate until the values of α and $\alpha_{0}$ converges. Finally, ${\hat{x}}_{\mathrm{MAP}}=\mu$.

### 2.3. Particle Swarm Optimization

PSO was inspired by the social behavior of bird flocking or fish schooling. PSO obtains a set of candidate solutions according to the mathematical formulas, which are based on the positions and velocities of the particles. These particles are iterated in the search space to solve the problem. The motion of each particle is not only affected by its local best known position, but is also guided toward the best known position in the search-space, which are updated by better positions found by other particles. PSO is a meta-heuristic algorithm (Algorithm 1) because it makes little or no assumptions about the problem being optimized and is able to search within a very large candidate solutions space.

Initially, each particle is randomly assigned with a position vector $x_{l}=\left[x_{l,1},x_{l,2},\ldots ,x_{l,D}\right]$ as well as a velocity vector $v_{l}=\left[v_{l,1},v_{l,2},\ldots ,v_{l,D}\right]$, where *D* is the dimension of a particle and l∈(1,2,…,S) with *S* representing the total particles number. Then each particle keeps track of its personal best position $P_{l}$ and the global best one G in the whole search space. After finding the two best fitness values for the *l*-th particle, it updates the position and velocity by the fourmulas

(10) $$\begin{array}{cc} v_{l}^{m+1} & =\omega^{m}v_{l}^{m}+a_{1}\left[r_{1,l}\left(P_{l}-x_{l}^{m}\right)\right]+a_{2}\left[r_{2,l}\left(G-x_{l}^{m}\right)\right],\\ x_{l}^{m+1} & =v_{l}^{m+1}+x_{l}^{m}. \end{array}$$

where *m* represents the current number of iteration, and $r_{1,l},r_{2,l}$ are random variables between [0,1]. $a_{1}$ and $a_{2}$ are the learning factors while ω is a weight factor that controls the velocity of the particle.


**Algorithm 1:** PSO algorithm **for** each particle **do**  initialize particle **end for** **while** target fitness or maximum epoch is not attained **do**  **for** each particle **do**   calculate fitness     **if** current fitness value better than (pbest) **then**    pbest = current fitness   **end if**  **end for**  set gbest to the best one among all pbest  **for** each particle **do**   update velocity   update position  **end for**  **end while**


## 3. Proposed System Model

**Figure 1:**
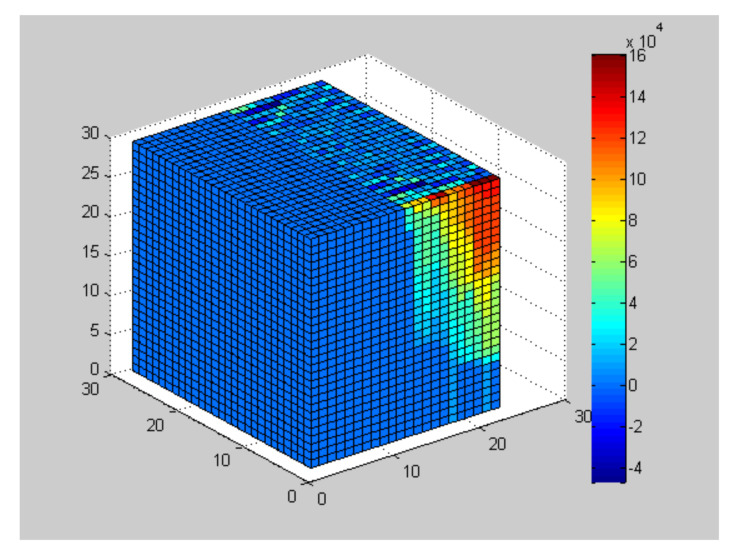
The segment-divided model of the 3-D network.

**Figure 2:**
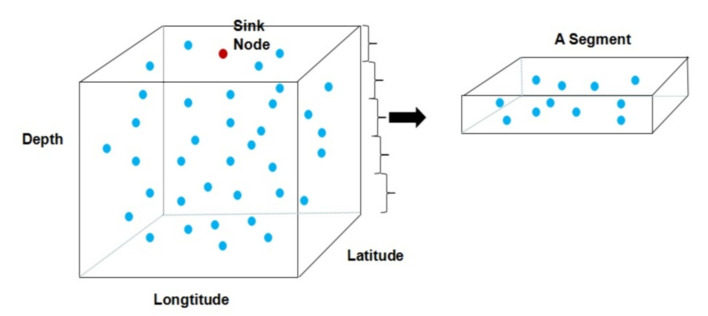
The FFT domain signal of temperature data.

We consider the scenario that sensor nodes are randomly deployed in a three dimensional undersea area and sink node (base station) is placed on top of the area as shown in Figure 1. As introduced before, CS can only be used when the signal has sparsity in some domain. In the next, we would prove that the monitored oceanic data such as temperature, hardness, and salinity are indeed sparse in Fourier transform domain because of their spatial correlation. Take the ocean underwater temperature data rendered by National Aeronautic and Space Administration (NASA) (https://www.nasa.gov/specials/ocean-worlds) for example, we transform the data into its fast Fourier transform (FFT) domain and the results validate the sparsity as show in Figure 2.

Another property of oceanic data is that its sparsity varies in different depth. As a result, we divide the network into different segments in line with its depth. Note that data with higher sparsity needs more information for recovery. Therefore, segments with higher sparsity are given higher cluster-heads chosen probability so that for these segments, more measurements are obtained. In our model, the data in different segments are transmitted to the sink node and reconstructed separately, hence every segment can be considered as a sub-model. The segment-divided network model is presented as Figure 1.

In reality, the data is collected in a 3-dimensional network. We need to ransform them into one-dimensional data in accordance with its horizontal position and depth first. Hence the data in the *i*-th segment is represented as $z_{i}=\left[z_{i,1},z_{i,2},\ldots ,z_{i,N}\right]$, where *N* is the total number of nodes in the *i*-th segment. However, for simplicity, in the sequel, we take every sub-model as an integral model and the data are regarded as $z=[z_{1},z_{2},\ldots ,z_{N}]$. Its sparse form is denoted as z=Ψx, where **x** is a sparse vector and Ψ is the FFT transform matrix.

**Figure 3:**
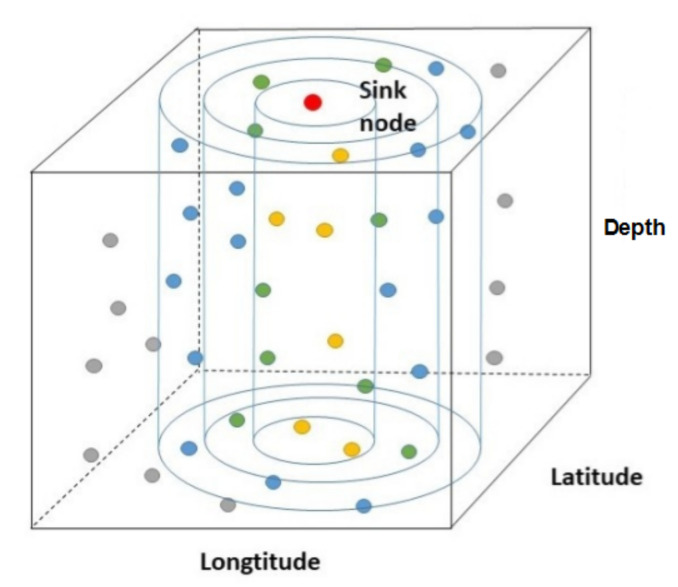
The layer-divided model of the 3-D network.

In each segment, we use CS to reduce the transmission burden to the sink node. Therefore, energy is saved and the network lifetime is prolonged. Clustering is also applied in our model to further economize on energy. The noisy measurement vector collected in the sink node is y=Φz+n=ΦΨx+n. For the purpose of making network energy-consuming balanced and easier for a node to find the next relay node, we divide all nodes into different layers according to their distances to the sink node. In the following work, we divided them into four distinct layers, those who are closer to the sink node are given smaller layer number, which is depicted in Figure 3.

## 4. Proposed Algorithm

The overall algorithm contains three main steps illustrated as follows.

**Figure 4:**
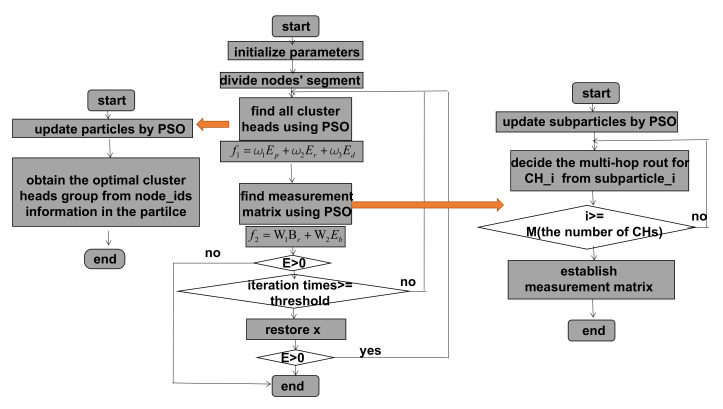
The flowchart of proposed algorithm.

Firstly, we use PSO algorithm to choose *M* CHs for each segment and divide the nodes in each segment into *M* clusters. The second step is to select one routing path for each cluster using PSO algorithm, hence we obtain *M* routs to compose an integral routing matrix $\Phi \in R^{M\times N}$. Figure 4 presents the flowchart of the whole procedure. In the flowchart, *E* represents the remaining energy of the whole network and $f_{1}$, $f_{2}$ are the fitness functions of clustering and routing steps respectively. $f_{1}$ considers energy consumption and uniformity of cluster head distribution while $f_{2}$ focuses on energy consumption state and BCRB.

The detailed description about how the CHs and routing paths, i.e., the measurement matrix are chosen will be presented in the next subsections. After we get the measurement matrix, the sink node receives a *M* dimensional measurement **y** and its measurement matrix Φ. Finally, at the sink node, we use BCS algorithm to reconstruct the sparse signal **x** and then transform it to the original temperature signal z. After obtaining signals for all the segments one by one, the base station merges them together to recover the complete original signal.

To design the final object, there are three important factors to be taken into consideration: the signal reconstruction accuracy, the remaining energy of the network, and the balance state of the network.

Usually, mean squared error (MSE) is used to measure the reconstruction quality of the signals. If $\hat{x}\left(y\right)$ denotes the recovered signal based on noisy measurements **y** represented by Equation (2). The MSE in estimation of the vector **x** is given by

(11) $$\mathrm{MSE}=E\left\{{∥x-\hat{x}∥}^{2}\right\}=Tr\left\{E\left[\left(x-\hat{x}\left(y\right)\right){\left(x-\hat{x}\left(y\right)\right)}^{T}\right]\right\}$$

However, the calculation of MSE requires the actual estimator which is impossible for us to get before choosing the measurement matrix. To solve this problem, we introduce Bayesian Cramér-Rao Bound (BCRB) to measure the lower bound of MSE; that is, MSE≥BCRB. Hence, adding BCRB into the fitness function is beneficial for us to find the solution with lower MSE, since minimizing BCRB is equal to obtaining minimum acceptable MSE. Subsequently, we will derive the formula of BCRB for our problem.

### 4.1. BCRB Derivation

The BCRB is computed as $BCRB=Tr\{J_{B}^{-1}\}$ with $J_{B}\in R^{N\times N}$ being the Bayesian Fisher information matrix (FIM) [42].

The i,j-th element of $J_{B}$, $J_{ij}$ is equal to $E_{XY}\left[\nabla_{x_{i}}\log p\left(x,y\right)\nabla_{x_{i}}^{T}\log p\left(x,y\right)\right]$. Herein, $\nabla_{v}≜\left[\frac{\partial }{\partial_{v_{1}}},\ldots ,\frac{\partial }{\partial_{v_{q}}}\right]$. Then $J_{ij}$ can be calculated as

$$\begin{array}{cc} J_{ij} & ≜E_{XY}\left[\nabla_{x_{i}}\log p\left(x,y\right)\nabla_{x_{j}}^{T}\log p\left(x,y\right)\right]\\ \; & =-E_{XY}\left[\nabla_{x_{i}}\nabla_{x_{j}}^{T}\log p\left(x,y\right)\right]\\ \; & =E_{XY}\left[\nabla_{x_{i}}\log p\left(y|x\right)\nabla_{x_{j}}^{T}\log p\left(y|x\right)\right]\\ \; & +E_{X}\left[\nabla_{x_{i}}\log p\left(x\right)\nabla_{x_{j}}^{T}\log p\left(x\right)\right]\\ \; & =-E_{XY}\left[\nabla_{x_{i}}\nabla_{x_{j}}^{T}\log p\left(y|x\right)\right]-E_{X}\left[\nabla_{x_{i}}\nabla_{x_{j}}^{T}\log p\left(x\right)\right]. \end{array}$$

Therefore, the Bayesian FIM is given by

(12) $$\begin{array}{cc} J_{B} & =-E_{XY}\left[\nabla_{x}\nabla_{x}^{T}\log p\left(y|x\right)\right]-E_{X}\left[\nabla_{x}\nabla_{x}^{T}\log p\left(x\right)\right]\\ \; & =-E_{\left(y,x\right)}\left\{\frac{\partial^{2}L\left(y|x\right)}{\partial x\partial x^{T}}\right\}-E_{\left(x\right)}\left\{\frac{\partial^{2}L\left(x\right)}{\partial x\partial x^{T}}\right\}. \end{array}$$

Firstly, we compute the second part of Equation (12). To solve L(x), we must obtain the density function of signal x in the first place. In our model, the sparse signal x follows the Gaussian distribution with a mean vector μ and a covariance matrix Σ. $\Sigma =\mathrm{diag}(\gamma_{1},\ldots ,\gamma_{N})$ is a diagonal matrix with $\gamma_{i}$ denoting the variance of $x_{i}$. As a result, the density of **x** is

(13) $$p\left(x;\gamma \right)=\prod_{i=1}^{N}{\left(2\pi \gamma_{i}\right)}^{-1/2}e^{-\frac{{\left(x_{i}-\mu \right)}^{2}}{2\gamma_{i}}}.$$

Hence $L\left(x\right)=\sum_{i=1}^{N}-\frac{1}{2}\ln2\pi \gamma_{i}-\frac{{\left(x_{i}-\mu \right)}^{2}}{2\gamma_{i}}$.

As the previous part is irrelevant to **x**, we abbreviate it as $\tilde{g}$. Then the formula in matrix form can be written as

(14) $$L\left(x\right)=\tilde{g}-\frac{1}{2}x^{T}\Sigma^{-1}x.$$

As a result $\frac{\partial^{2}L\left(x\right)}{\partial x\partial x^{T}}=\Sigma^{-1}$.

Secondly, we turn to deal with the first part of FIM. To obtain $-E_{\left(y,x\right)}\left\{\frac{\partial^{2}L\left(y|x\right)}{\partial x\partial x^{T}}\right\}$, we need to know p(y|x). Since y=ΦΨx+n, the probability density function of **y** is related to the noise term n given the information of **x**. The noise can be the Gaussian distribution; that is, $p\left(n\right)=\prod_{i=1}^{N}{\left(2\pi \sigma^{2}\right)}^{-1/2}e^{-\frac{n_{i}^{2}}{2\sigma^{2}}}$. Hence, we have

(15) $$p\left(n\right)={\left(2\pi \sigma^{2}\right)}^{-N/2}e^{-\frac{{∥y-\Phi \Psi x∥}^{2}}{2\sigma^{2}}}.$$

Subsequently,

$$\begin{array}{cc} p(y|x) & =-\frac{N}{2}\times \log2\pi \sigma^{2}-\frac{{∥y-\Phi \Psi x∥}^{2}}{2\sigma^{2}}\\ \; & =-\frac{N}{2}\log2\pi \sigma^{2}-\frac{1}{2\sigma^{2}}{\left(y-\Phi \Psi x\right)}^{T}\left(y-\Phi \Psi x\right), \end{array}$$

and then

(16) $$E_{\left(y,x\right)}\{\frac{\partial^{2}L\left(y|x\right)}{\partial x\partial x^{T}}\}\;=-\frac{1}{\sigma^{2}}\Psi^{T}\Phi^{T}\Phi \Psi .$$

Finally, we put the two parts together as the description of FIM,

$$\begin{array}{cc} J_{B} & =-E_{\left(y,x\right)}\left\{\frac{\partial^{2}L\left(y|x\right)}{\partial x\partial x^{T}}\right\}-E_{\left(x\right)}\left\{\frac{\partial^{2}L\left(x\right)}{\partial x\partial x^{T}}\right\}\\ \; & =\frac{1}{\sigma^{2}}\Psi^{T}\Phi^{T}\Phi \Psi +\Gamma^{-1} \end{array}$$

Therefore, in our model, BCRB is represented by $Tr\{\frac{1}{\sigma^{2}}\Psi^{T}\Phi^{T}\Phi \Psi +\Gamma^{-1}\}$.

### 4.2. Underwater Energy Consumption Model

Ref. [43] gives an in-depth analysis of the energy consumptions of underwater acoustic networks according to the conceptions of underwater acoustic channel attenuation model, noise model and bandwidth model. The total energy consumption associated to one hop (which includes both the transmit and the receive energy at the two ends of the link), is determined by the power $P_{r}$, acoustic electric conversion power $P_{t}^{el}\left(l\right)$ which is the power required to transform acoustic signal to electric signal and single hop transmission delay $t_{hop}\left(L,l\right)$. Specifically, the energy consumed by each single hop, $E_{hop}$ is calculated as:

(17) $$E_{hop}\left(L,l\right)=t_{hop}\left(L,l\right)\times \left(P_{r}+P_{t}^{el}\right).$$

The single hop transmission delay $t_{hop}\left(L,l\right)$ is determined by package length *L* and the distance *l* between two transmitting nodes as well as channel effective bandwidth αB(l) where α means channel utilization rate. So the formula becomes

(18) $$E_{hop}\left(L,l\right)=t_{hop}\left(L,l\right)\times \left(P_{r}+P_{t}^{el}\right)=\frac{L}{\alpha B(l)}\times \left(P_{r}+P_{t}^{el}\right)$$

$P_{t}^{el}\left(l\right)$ is calculated as

(19) $$P_{t}^{el}\left(l\right)=\frac{P_{t}\left(l\right)\times 10^{-17.2}}{\eta }.$$

Herein, $10^{-17.2}$ is the conversion factor from acoustic power in dBreμPa to electrical power in Watt, η represents electronic circuit conversion efficiency and $P_{t}\left(l\right)$ is the transmitting power of underwater nodes, which is calculated as

(20) $$P_{t}\left(l\right)=B\left(l\right)\times A\left(l,f_{0}\left(l\right)\right)\times N\left(f_{0}\left(l\right)\right)\times SNR_{tgt}.$$

N(f(l)) and A(l,f(l)) are noise parameter and attenuation coefficient, respectively. $AN=A\left(l,f(l)\right)N\left(f(l)\right)$ is the channel parameter and can be plotted as a curve in terms of *f* for given *l*. Therefore, for certain *l*, we can obtain its best frequency $f_{0}\left(l\right)$ and then find its corresponding AN product, $N\left(f_{0}\left(l\right)\right)\times A\left(l,f_{0}\left(l\right)\right)$ from the AN curve.

$SNR_{tgt}$ denotes the target signal noise rate for the terminal to receive signals correctly. B(l) is the usable bandwidth given $SNR_{tgt}$, which is $B\left(l\right)=b\times l^{-\beta }$. The positive parameters b,β depend on the target SNR as well. Unlike the transmit power, the receive power $P_{r}$ is independent of distance, and rather depends on the complexity of the receive operations. So it could be set as a constant value. More details can be found in [44].

In conclusion, the energy consumed for each hop in the terms of package length *L* and distance *l* is calculated as:

(21) $$\begin{array}{cc} \; & E_{hop}\left(L,l\right)\\ \; & =t_{hop}\left(L,l\right)\times \left(P_{r}+P_{t}^{el}\right)\\ \; & =\frac{L}{\alpha B(l)}\times \left(P_{r}+\frac{B\left(l\right)N\left(f_{0}\left(l\right)\right)A\left(l,f_{0}\left(l\right)\right)SNR_{tgt}\times 10^{-17.2}}{\eta }\right) \end{array}$$

Based on such a model, we can calculate the energy consumed by each node which participates the routing.

### 4.3. PSO for Clustering

First we use PSO to find appropriate nodes to be CHs. Because we divided the network into different segments, the percentage of CHs within each segment is highly related to the sparsity of the segment. In general, we set the CH ratio *q* to be 0.4, only in some segments which have relative low sparsity, the value of $q_{i}$ increases slightly in a trend like $q_{i}=0.4+0.05\times \left\lceil s_{i}-0.08\right\rceil$ where $s_{i}$ is the sparsity of the *i*th segment.

The particle’s dimension is the same as the number of CHs. To initialize the particle, we generate *M* random integer numbers within [1,N]. For example, the *i*-th particle $P_{i}$ is *M* dimensional and $P_{i,m}$ represents the *m*-th value of it. If $P_{i,m}=25$, it means that in this particle, we choose node 25 to be the *m*-th CH. As a node cannot be chosen repeatedly in one particle, if a particle has duplicated numbers, it will be assigned a penalty fitness value −1 to be excluded during later progress.

After initializing all the particles, we calculate the fitness value for each particle. According to their fitness value, we choose the local best and the global best particles, update the particle position which corresponds to the coordinates of the nodes in this particle and velocity to get new fitness values.

In this part, we mainly focus on energy consumption and uniformity of cluster head distribution, hence the fitness function *f* does not include the measurements for signal recovery qualities in current stage. The fitness function $f_{1}$ comprises of three parts as below:

- $E_{p}$, represents the energy evaluation factor and $E_{p}$ is given by
  (22) $$E_{p}=\frac{\sum_{m=1}^{M}E_{res_{m}}}{\sum_{i=1}^{N}E_{init_{i}}},$$
  which measures the remaining energy of the chosen CHs.
  Note that $E_{init_{i}}$ is the initial energy of the *i*-th node while $E_{res_{m}}$ is the residual energy of the *m*-th node and equals to $E_{ini_{m}}-E_{c_{m}}$, and
  $$\begin{array}{cc} E_{c_{m}}= & \left\{\begin{array}{c} 0\;\;\mathrm{if}\mathrm{node}m\mathrm{was}\mathrm{not}\mathrm{involved}\mathrm{in}\mathrm{the}\mathrm{routing}\\ t_{hop}\left(L,l_{m,r}\right)\times P_{r}+t_{hop}\left(L,l_{m,t}\right)\times P_{t}^{el}\;\;\mathrm{if}\mathrm{node}m\mathrm{was}\mathrm{involved}\mathrm{in}\mathrm{the}\mathrm{routing}, \end{array}\right. \end{array}$$
  where $l_{m,r}$ measures the hop length when node *m* is the receiver and $l_{m,t}$ measures the hop length when node *m* is the transceiver.
  We tend to choose nodes with higher residual energy to be CHs due to the fact that cluster heads consume more energy than normal nodes.
- $E_{c}$ indicates the evaluation factor for the intra-cluster compactness and measures the average distance between nodes and their cluster heads.
  We calculate
  $$f=\max_{m=1,2,\ldots ,M}\sum_{\exists n_{i}\in C_{m}}d_{i,CH_{m}}/\left|C_{m}\right|,$$
  which measures the maximum average Euclidean distance between nodes and their CHs. $d_{i,CH_{m}}$ measures the distance between node *i* and $CH_{m}$. $|C_{m}|$ is the number of nodes that belong to cluster $C_{m}$. Our aim is to minimize *f* because the nodes are closer to its cluster heads with smaller *f*. Therefore, $E_{c}=1/f$.
- $E_{e}$ is the evaluation factor of the uniformity of CH distribution. It measures the uniformity of CH distribution. Firstly we calculate all nodes’ distances to each other. $D=d_{1},\ldots ,d_{N}$ where $d_{j}=d_{j,1},\ldots d_{j,i},\ldots ,d_{j,N},i\neq j$ and $d_{j,i}$ measures the distance between node *j* and node *i*. In an unevenly distributed network, the sum of D must be higher than relatively evenly distributed network. As a consequence, we add this value $E_{e}=\sum_{j=1}^{M}d_{j}/\left|C_{m}\right|$ on our fitness function.

To sum up, the final form of fitness function $f_{1}$ of our cluster choosing algorithm is given by:

$$\begin{array}{cc} f_{1} & =w_{1}E_{p}+w_{2}E_{c}+w_{3}E_{e},\\ \; & \mathrm{where}\left\{\begin{array}{c} w_{1}+w_{2}+w_{3}=1\\ w_{i}\geq 0,i\in \left\{1,2,3\right\} \end{array}\right. \end{array}$$

and $w_{i}$ is the weighting factor for the *i*-th item. To think about the problem in a balanced way, $w_{1}$ and $w_{2}$ are randomly chosen from the range [0.2,0.4] while $w_{3}=1-w_{1}-w_{2}$.

When a particle contains duplicated nodes, we set a penalty factor to it to exclude it from iterations.

This iteration keeps going on until either the global best fitness value is lower than the threshold we set or the maximal iterative times is reached. The global best particle obtained by the last iteration is the output of the cluster head selection scheme.

### 4.4. PSO for Choosing Routing Paths

The final step of our algorithm is to find the routing paths, then the measurement matrix. PSO is used in this stage as well and herein each particle contains *M* sub-particles, where *M* is the number of CHs. A sub-particle includes the nodeID information while sub-particle position includes priorities corresponding to all the nodes denoted in the sub-particle.

Each sub-particle determines a single rout from each cluster to the base station. Since we have already chosen all the CHs, there are two steps left for each CH to fulfill the corresponding routing path: (1) Each CH chooses the nodes in its cluster which transmit the messages to it in one hop; (2) After collecting messages in its cluster, each CH starts a multi-hop rout to the base station.

In one word, a complete routing path contains two parts, the one-hop inner cluster part and the multi-hop outer part. Therefore we divide the sub-particle into two parts, *a* and *b*. Part *a* determines which nodes from the inner cluster transmit their data to the CH while part *b* finds the routing path from the CH to the base station. It is difficult to jointly optimize these two part. Instead, we construct inner cluster part and outer cluster part separately.

**Figure 5:**
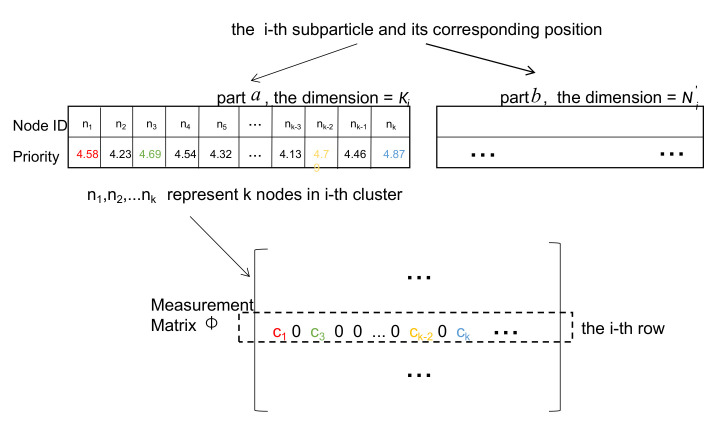
The demo for mapping part *a* of sub-particle *i* into Φ, where $c_{j}$ denotes random measurement coefficient.

The dimension of part *a* of sub-particle *i* equals to $K_{i}$, where $K_{i}$ is the node numbers of each cluster except the *i*-th CH. The sub-particle *i* selects $\epsilon \times K_{i}$ nodes with highest priorities from part *a* to transmit data to the *i*-th CH in one hop. ϵ is the ratio controlling parameter which we would optimize in the simulation section. Figure 5 gives an example on how to choose nodes within one cluster and how to map part *a* of a sub-particle into real measurement matrix. The measurement matrix with dimension M×N is initially set to all zero matrix, where *M* is the number of CHs and *N* is the total number of nodes. Once a node is chosen into *i*-th rout, its corresponding position in the *i*-th row of measurement matrix is set to a randomly distributed coefficient which obeys Gaussian distribution.

The algorithm to find a multi-hop routing path from the *i*-th CH to the base station from part *b* is more sophisticated and will be illustrated in detail in the following.

- **Choosing candidate nodes**

Regarding that if we take all nodes into consideration for each rout, the dimension of a sub-particle would be too large. Given the fact that when a node try to find its rout, those nodes in the opposite area from sink node may never be included. Therefore, before the initialization, we calculate the distances of all nodes to the sink node $Dis_{s}$ and the distance of all nodes to the CHs $Dis_{c}$. Then choose an appropriate group of candidate nodes for each CH first. $Dis_{s}=d_{s,1},\ldots ,d_{s,N}$ represents nodes’ distances to the sink node while $Dis_{c}=d_{c,1},\ldots ,d_{c,N}$ represents nodes’ distances to the CH. $dis=\sqrt{{\left(x_{s}-x_{c}\right)}^{2}+{\left(y_{s}-y_{c}\right)}^{2}+{\left(z_{s}-z_{c}\right)}^{2}}$ is the distance between the CH and sink node where $x_{s},y_{s},z_{s}$ represent the coordinates of the sink node and $x_{c},y_{c},z_{c}$ are the coordinates of the CH. Note that these coordinates values could be obtained by range-based or range-free localizations before the data transmission. Only when a node *k* satisfies $d_{s,k}\leq dis$ and $d_{c,k}\leq dis$, it would be added to the candidate group. Once the candidate groups are chosen, they will not be altered during the whole iteration period. Suppose the size of candidate group for the *i*-th CH is $N_{i}^{\prime }$, then the dimension of part *b* of the sub-particle *i* equals to $N_{i}^{\prime }$.

ii. **Initialization**

To find part b, we make use of the network’s layered structure as we need to take directions into consideration. The nodes that are in the closer layer to the base station ought to have higher priority. This layer-based initialization method can control the routing path’s direction towards the sink node as well as avoid trapping into a dead cycle and accelerate iterative process.

The priorities of the nodes in the farthest layer are initialized by randomly generating numbers between 0 to 1. In our layered model, the fourth layer is the farthest layer, so the priorities of the nodes in the fourth layer are set from 0 to 1, while the priorities of the nodes in the third layer are from 1 to 2. In one word, when the number of the layer decreases by 1, the initial priorities of the nodes increase by 1. Hence the priority is not initialized completely randomly but related to the layer the node belongs to.

**Figure 6:**
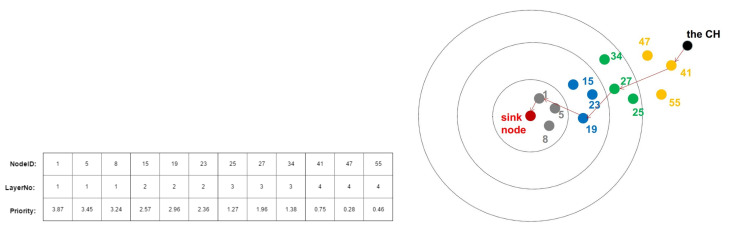
An example of hierarchical priorities initialization for part *b* and its corresponding topological routing path in the cross-section view.

Figure 6 shows an example of our initialization. In this example, for the CH, there are altogether 12 candidate nodes dispersed in 4 different layers. It can be easily observed that the nodes in the higher layers have lower initialized priorities. That is how we achieve hierarchical initialization and obtain initialized multi-hop routing path from the CH to the sink node.

iii. **Iteration**

After initializing the particles, in each iteration, we are going to calculate particles’ fitness values in terms of measurements matrices and select local best and global best ones. Then the particle positions are updated.

We have already introduced how to map part *a* of the sub-particle *i* to the corresponding row. Here we introduce how we map part *b* of sub-particle *i* to the *i*-th row of the measurement matrix.

Firstly we set the corresponding position of the *i*-th CH in the *i*-th row of Φ to a random Gaussian distributed coefficient. Then we find the next relay node by two steps iteratively until the complete routing path is found.

Step 1: If the previous node’s distance to the sink node is smaller than its transmission range $R_{c}$, stop searching since we already obtain a single rout from the *i*-th CH to the sink node. Otherwise, we go to step 2.

Step 2: Find all nodes that are not only in the candidate group but also within the previous node’s transmission range, compare their priorities and find the largest one as the next relay node. Meanwhile, we set the corresponding position of chosen relay node in the *i*-th row of Φ to a random Gaussian distributed coefficient.

After mapping *M* sub-particles to *M* rows, the measurement matrix Φ is determined. The generated Φ is then used to compute the fitness value of corresponding particle. The fitness function includes two main items, the measurement of reconstruction quality, $B_{r}$, which is related to BCRB and the measurement of energy consumed, $E_{s}$.

As we calculated before, the formula of the BCRB is given by

(23) $$\mathrm{BCRB}=Tr\{\left(\frac{1}{\sigma^{2}}\Psi^{T}\Phi^{T}\Phi \Psi +{\hat{\Gamma }}^{-1}\right)\}.$$

As BCRB implies a theoretical lower bound of reconstruction’s mean square error, we wish it to be as small as possible. So $B_{r}=1/Tr\left\{\left(\frac{1}{\sigma^{2}}\Psi^{T}\Phi^{T}\Phi \Psi +{\hat{\Gamma }}^{-1}\right)\right\}$.

The energy consumption of the routings we chose is measured by $E_{s}$. It consists of three factors like

(24) $$E_{s}=1/E_{r}+E_{l}+E_{b}.$$

The first item $E_{r}=\sum_{k=1}^{N}\frac{E_{c_{k}}}{E_{res_{k}}}$, where $E_{c_{k}}$ is the energy consumed at this round by node *k* and $E_{res_{k}}$ is the rest energy of this node. It measures the energy cost ratio of nodes and should be small considering the durability of a network.

Besides $E_{r}$, $E_{l}=\sum_{k=1}^{N}\frac{E_{res_{k}}}{E_{init_{k}}}$, where $E_{init_{k}}$ is the initial energy of the *k*-th node.

On top of that, the balance of this network also needs to be considered. A well-known fact is that the nodes near the sink node are prone to consume more energy than the nodes far away. Those nodes burden heavier transmission tasks and are easier to convey their data to the sink repeatedly because they are more likely to be relay nodes. So to obtain a balanced network, we introduce a factor $E_{b}$ to describe a energy consumption ratio of different layer. $E_{b}=E_{1}/E_{4}+E_{2}/E_{3}$, where $E_{i}$ represents the remaining energy of the *i*- th layer. $E_{b}$ should be as large as possible as the lower layer tends to remain more energy in a balanced network than in an uneven network.

In a conclusion, the final fitness function consists of two parts as below,

(25) $$f_{2}=W_{1}B_{r}+W_{2}E_{s}$$

where $W_{i}$ is a weighting factor and $W_{1}+W_{2}=1,W_{i}\geq 0,i\in \left\{1,2\right\}$. In the simulation, $W_{1}$ is chosen randomly from the range [0.4,0.6] while $W_{2}=1-W_{1}$.

## 5. Simulation Results

We use the real ocean temperature data provided by NASA (https://www.nasa.gov/specials/ocean-worlds) to test our algorithm against the two cutting-edge methods. Note that the sensor network they deploy to monitor the environment is huge and we only choose 1000 nodes from this network which are deployed in a 3-D undersea region divided into 10×10×10 grids. The size of the area is 18.5 km × 14.5 km × 0.8 km. We compare our method with other two cutting edge method which also applies CS into routing. The method introduced in [31] for 3D UWSN is named as “DRMCS” which is the abbreviation of “distributed random multi-hop compressive sensing”. The other method is called “ICCS” following its name in [29] and later on in [30]. We extend it to 3D scenario by changing the clustering and routing methods to 3D protocol through replacing the 2D distance (x,y) to 3D distance (x,y,z). The parameters we set for the energy consumption model in Section 4.1 are listed in Table 1 according to [43].

Before the whole simulations, we firstly select the appropriate value of parameter ϵ. It is impossible for us to obtain the signal **z** before the algorithm is run. To solve this problem, we do some preparatory experiments to decide an appropriate ϵ. In the first five rounds, we set ϵ=0.9, run the experiments once, and then we obtain the reconstructed signal $\hat{z}$. The reconstructed signal $\hat{z}$ is regarded as the approximation of the original signal z in order to compute the reconstruction error in the following simulations. That is, afterwards we ran the first five rounds with different ϵ ranging from 0.2 to 0.8, respectively. The average consuming energy and the reconstruction error are compared. For example, the results of round 1, 3 and 5 are shown in Table 2. Considering the reconstruction error and energy consumption state integrally, we found that in our algorithm ϵ=0.4 is the most suitable. As a result, for the rest rounds, ϵ is set to be 0.4.

All experiments were performed on MATLAB 2018b and a computer with intel(R) Core(TM) i7-8700K 2.50 GHz CPU, 16.00 GB RAM and the Windows 10 system.

**Figure 7:**
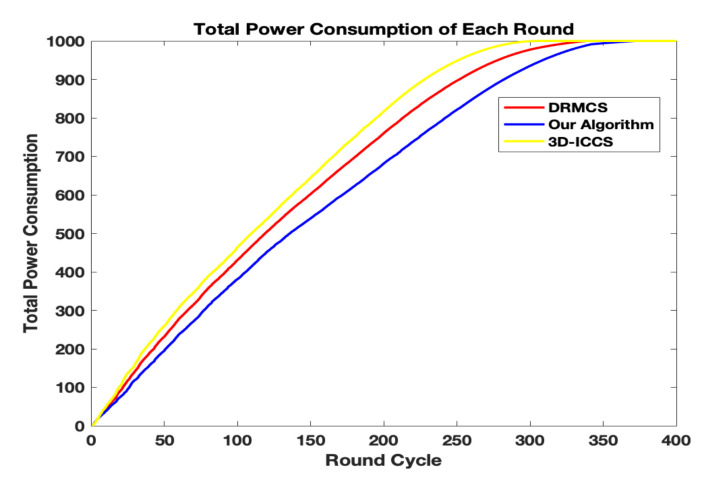
The comparison results in terms of total energy consumption.

**Figure 8:**
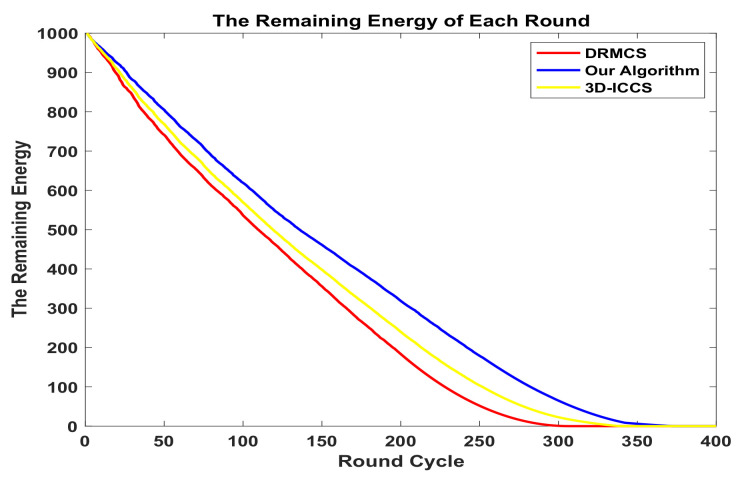
The comparison results in terms of remaining energy.

In Figure 7 and Figure 8, we compare the total energy consumption and the remaining energy for each round while the measurement ratio M/N is set to 0.4. Obviously our algorithm survives longer than the other two state-of-the-art methods.The first tier utilizes PSO to find the best cluster heads group while the second tier utilizes PSO to find the best routing matrix so that the whole network has the lowest average consuming energy per cycle while maintaining the sensing error in an acceptable range. Note that in the second tier, the optimization of the routing path can not only reduce the average energy cost per round but also help to make the network more balanced. In other words, besides the fact that our PSO-based routing scheme consumes less energy each round, another important reason for longer network lifetime is that our scheme obtains more balanced energy-consumption condition.

**Figure 9:**
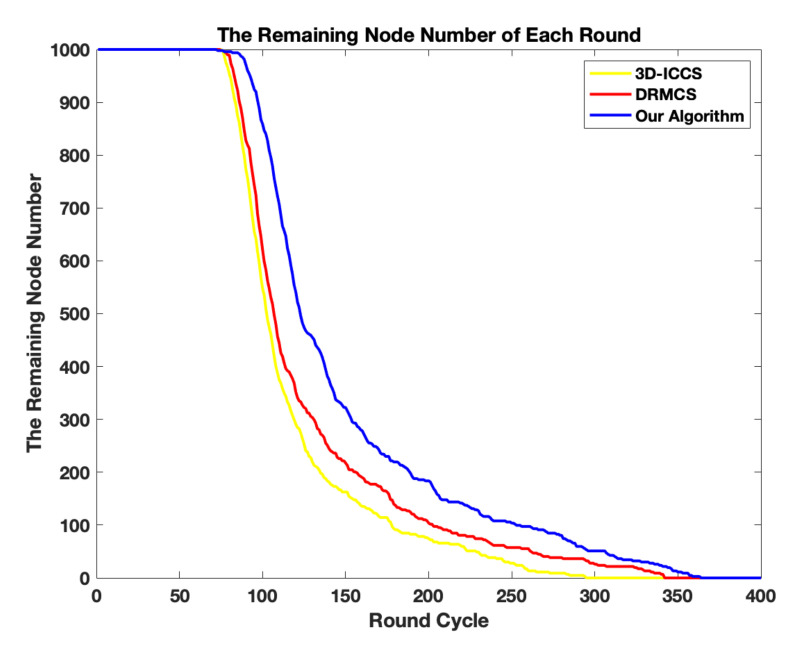
The comparison results in terms of remaining living nodes.

The more balanced condition and less energy-consuming phenomenon of our scheme can also be proved in Figure 9, which shows the remaining living nodes of the network in each round. From Figure 9, we can observe that by our algorithm, the first dead node appears much later. The proposed algorithm has its first dead node at round more than 100 while the other algorithms has its first dead node at about round 70. Such a fact indicates that the proposed two-tier protocol consumes less energy each round as well as consumes energy more equally for each node of the whole network. The improvement attributes to the fact that in order to better balance the whole network, we add a factor named “network-balancing” in the particles’ fitness value calculation. Considering that the nodes closer to the sink burden heavier transmission load and hence are easier to die, we put energy consumption ratios of inner and outer layers into consideration. Reducing the ratio can avoid repeatedly choosing inner layer nodes and balance the network energy consumption better.

**Figure 10:**
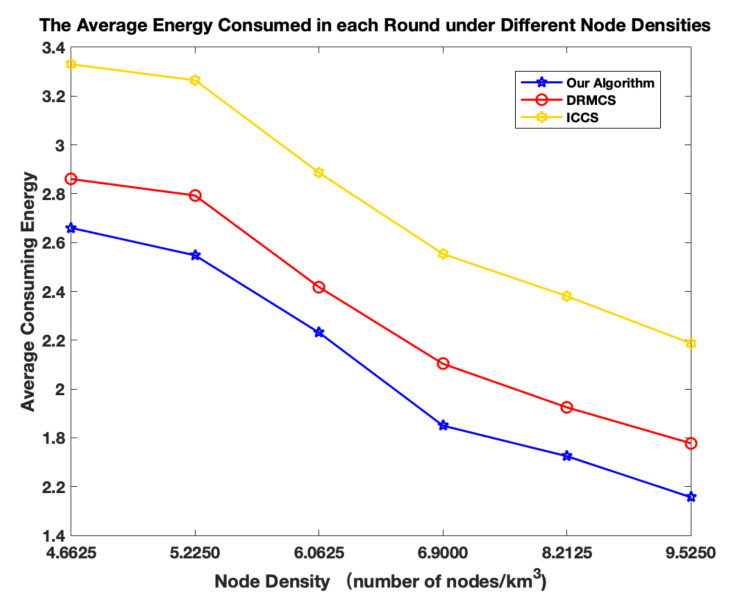
The comparison results in terms of average energy consumption with different node densities.

**Figure 11:**
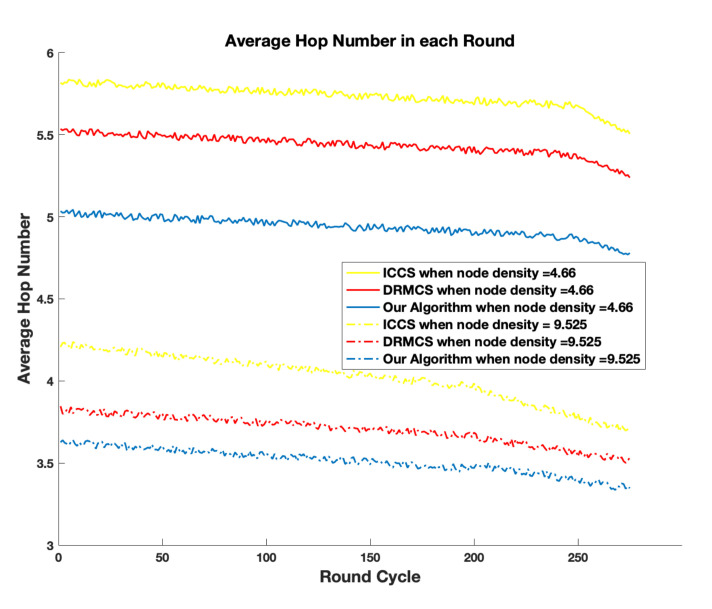
The comparison results in terms of average hop numbers with two different node densities.

In order to observe the impact of node density on the network performance, we fix the total node number as 1000 and vary the volume of the area where these nodes are deployed. As shown in Figure 10, the average energy consumption in each round decreases with the increase of node density. Since the node number is fixed, the initial energy is fixed. Hence lower average energy consumption in each round means larger round cycles. This is due to the fact that we shrink the volume so that the average distances from CHs to sink node are shortened. From Figure 10, it can also be observed that compared with other two schemes, our algorithm consumes less average energy each round in the whole range of node density. To measure the delay of relevant schemes, we calculate the average hop numbers in the case of two different node densities. Average hop number is obtained by $\overset{M}{\underset{i=1}{\Sigma }}hop_{i}/M$, where $hop_{i}$ measures the hop numbers from the *i*-th CH to the sink node. Obviously, since the total node number is fixed and the volume is decreased, average hop numbers, i.e., delay is lower with higher node density as shown in Figure 11. On the other hand, our algorithm always presents the lowest delay compared with relevant schemes.

**Figure 12:**
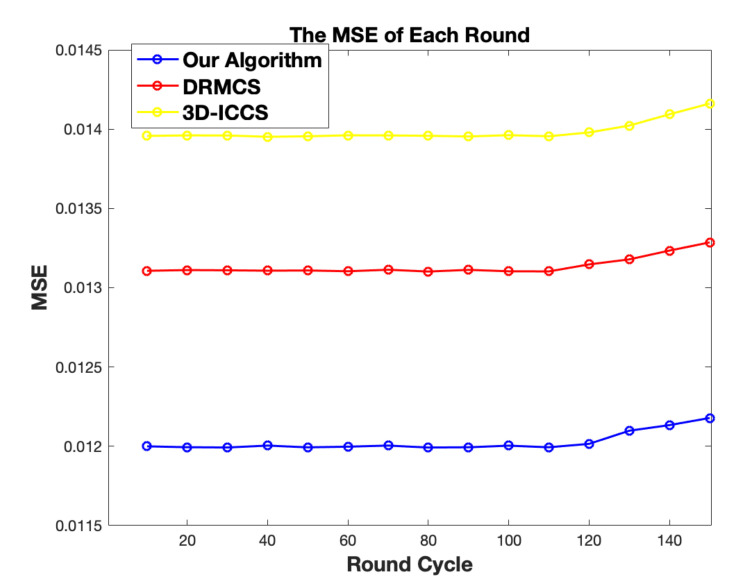
The comparison results in terms of data recovery qualities.

Another performance we compare is the sensing quality which is measured by MSE between the original signal and reconstructed error. It can be observed from Figure 12 jointly with Figure 8 that our proposed algorithm tends to build a matrix Φ that leads to the smallest sensing error and meanwhile the energy consumed by our algorithm is the least in each round.

**Figure 13:**
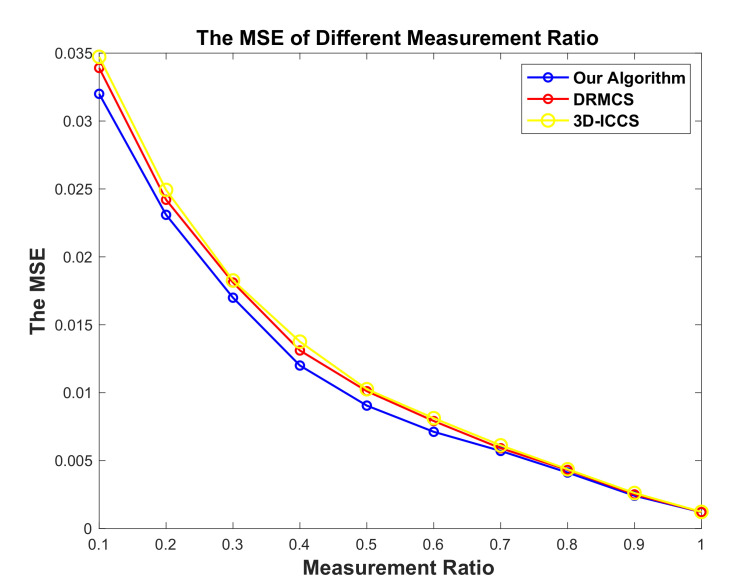
The comparison results in terms of MSE in different measurement ratio.

Besides the previous experiments, we vary the measurements ratio M/N as well to observe the MSE with varying ratio. We test the average MSE for first 20 rounds. It can be observed that the MSE decreases with the increase of measurement ratio for each algorithm. Figure 13 validates that our algorithm presents significant advantage when the measurement ratio is less than 0.5. The reason is that we add the factor $B_{r}$, which considers the reconstruction quality, into the fitness function while performing PSO for routing. Our proposed algorithm works well to reduce the MSE when the measurement ratio is low. While the measurement ratio goes up, the superiority of our proposed algorithm becomes less significant.

## 6. Conclusions and Future Work

In this paper, we proposed a two-tier Particle Swarm Optimization (PSO) algorithm in the clustering and routing stages for distributed multi-hop compressed sensing in 3-D UWSN. We divided the whole network into different segments according to the vertical distances of the nodes. On top of that, we also divided the whole network into different layers according to the horizontal distances from the nodes to the sink and the PSO algorithm we apply in the network is firmly associated with the layers. Dividing the layers help to define the energy consumption in different layers to measure energy-consuming balance and to initialize the particle hierarchically so as to accelerate the particle convergence speed. And a factor to forecast the signal recovery performance is added on the fitness function of the PSO algorithm for routing to control the MSE of the reconstructed signal while maintaining a relatively long lifetime. Our proposed algorithm is compared with recently proposed algorithms for distributed multi-hop compressive sensing routing problem using real marine temperature data rendered by NASA. The results validate that our proposed algorithm has a longer life time with lower measurement error and postpones the round that has the first dead node.

For the future work, we will focus on the synthesized consideration of the clustering and routing stage while doing optimization. In the current study, the two stages are considered separately. On the further study, we will conduct the two steps consistently to improve the performance a step further. 

## Figures and Tables

**Table 1:** Energy Consumption Parameters.

Energy Consumption Parameter	Values
η	0.5
α (bps/Hz)	0.5
*k*	1.5
$SNR_{tgt}$ (dB)	8
$P_{r}$ (W)	2
*b* (dB re kHz)	14.39
β (dB re kHz/km)	−0.55
$R_{c}$ (km)	3.5

**Table 2:** Performances by Different ϵ for round 1, 3, 5.

ϵ	Energy Consumption	Reconstruction Error
0.2	3.164	0.01419
0.4	3.643	0.01201
0.6	4.445	0.01249
0.8	5.050	0.01240
ϵ	**Energy Consumption**	**Reconstruction Error**
0.2	3.157	0.01421
0.4	3.655	0.01203
0.6	4.426	0.01251
0.8	5.039	0.01242
ϵ	**Energy Consumption**	**Reconstruction Error**
0.2	3.168	0.01437
0.4	3.637	0.01208
0.6	4.439	0.01257
0.8	5.012	0.01248
